# Repurposed drugs in combinations exert additive anti-chikungunya virus activity: an in-vitro study

**DOI:** 10.1186/s12985-023-02271-0

**Published:** 2024-01-04

**Authors:** Kusuma Sai Davuluri, Rajnandini Ghanghav, Gunwant Ahire, Mahadeo Kakade, Sarah Cherian, Kalichamy Alagarasu, Deepti Parashar

**Affiliations:** 1 Dengue & Chikungunya Group, ICMR-National Institute of Virology, 20-A, Dr. Ambedkar Road, Pune, Maharashtra 411001 India; 2 Bioinformatics and Data Management Group, ICMR-National Institute of Virology, 20-A, Dr. Ambedkar Road, Pune, Maharashtra 411001 India

**Keywords:** Chikungunya virus (CHIKV), Drug repurposing, Combination of repurposed drugs, Synergistic effect, In silico screening, In vitro validation

## Abstract

Chikungunya virus (CHIKV) infection causes chikungunya, a viral disease that currently has no specific antiviral treatment. Several repurposed drug candidates have been investigated for the treatment of the disease. In order to improve the efficacy of the known drugs, combining drugs for treatment is a promising approach. The current study was undertaken to explore the antiviral activity of a combination of repurposed drugs that were reported to have anti-CHIKV activity. We explored the effect of different combinations of six effective drugs (2-fluoroadenine, emetine, lomibuvir, enalaprilat, metyrapone and resveratrol) at their non-toxic concentrations against CHIKV under post infection treatment conditions in Vero cells. Focus-forming unit assay, real time RT-PCR, immunofluorescence assay, and western blot were used to determine the virus titre. The results revealed that the combination of 2-fluoroadenine with either metyrapone or emetine or enalaprilat exerted inhibitory activity against CHIKV under post-infection treatment conditions. The effect of these drug combinations was additive in nature compared to the effect of the individual drugs. The results suggest an additive anti-viral effect of these drug combinations against CHIKV. The findings could serve as an outline for the development of an innovative therapeutic approach in the future to treat CHIKV-infected patients.

**Supplementary Information:**

The online version contains supplementary material available at 10.1186/s12985-023-02271-0.

## Introduction

Chikungunya is a member of the Togaviridae family’s Alphavirus genus. The virus causes outbreaks of acute febrile polyarthralgia and is primarily transmitted by mosquitoes, particularly *Aedes aegypti* and *Aedes albopictus*. As of 2021, India was experiencing a surge in chikungunya cases. National Vector Borne Disease Control Programme (NVBDCP) reported more than 65,000 suspected cases of chikungunya in India in 2021 (https://ncvbdc.mohfw.gov.in/), assessed on 17.05.2023). As of now there is no established drug or vaccine developed against the CHIKV. Repurposing existing medications is one of the strategies which can be used to combat viral diseases, and it also provides insights for future viral threats and drug discovery approaches. Our previous studies have supported the possibility of repurposing existing drugs against CHIKV infection [1]. By targeting different stages of the virus life cycle, the overall efficacy of treatment can be increased and the risk of drug resistance can be reduced. Moreover, it enables reducing the dose of individual drugs and thereby lessens the side effects caused by the drugs. The combination of drugs can produce synergistic or additive antiviral effects. This approach is powerful and could lead to more effective viral suppression and faster recovery [2]. Targeting both virus entry and replication offers potential for synergistic combinations. Combination antiviral treatments are established for several chronic RNA virus infections. Our recent findings showed six repurposed drugs, 2-fluoroadenine (2-FA), lomibuvir (L), emetine (EM), enalaprilat (EN), metyrapone (M), and resveratrol (R) to exhibit in vitro anti-CHIKV activity [1]. Several compounds have been investigated for their diverse properties and therapeutic potential. 2-Fluroadenine (2FA), carbocyclic, and acyclic nucleoside analogues have been identified as antitumor and antiviral agents [3]. Resveratrol (R) is recognized for its antioxidant properties [4] and has shown antiviral activity against HCoV-229E and SARS-CoV-2 coronaviruses, as well as oncogene inhibition and geroprotective effects [5–7]. Metyrapone is utilized to control hypercortisolism in Cushing’s syndrome and also possesses antidepressant properties while reducing allergic inflammation [8, 9]. Enalaprilat functions as a postoperative hypertension controller and demonstrates antiviral activity by inhibiting the interaction of ACE2 (Angiotensin converting enzyme 2) and the SARS-CoV-2 spike protein [10]. Lomibuvir acts as an inhibitor of hepatitis C virus NS5B polymerase and dengue virus-2 [11, 12]. Felbinac is used for the treatment of localized extra-articular rheumatic diseases and soft tissue injuries [13, 14]. Emetine alkaloids have been investigated for their potential use in treating SARS-CoV-2, Zika, and Ebola virus infections, as well as their anticancer effects in U2OS human osteosarcoma [15–17]. These drugs have demonstrated various properties such as antioxidant, antiviral, anti-inflammatory, cardioprotective, antidepressant, and immunosuppressive effects [3, 5, 9, 11, 16, 18, 19]. In the present study, we explored the antiviral activity of various combinations of the aforesaid compounds as candidate anti-CHIKV drug combinations for repurposing.

## Materials and methods

### Cells & virus

We employed the Vero CCL-81 cell line (ATCC® CCL 81™), derived from African green monkey kidney, to evaluate antiviral efficacy. The cells were cultivated at 37 °C with 5% CO_2_ in Minimal Essential Medium (MEM) supplemented with 10% fetal bovine serum (FBS) (GibcoTM, Grand Island, NY, USA), and an antimycotic antibiotic solution (Sigma Aldrich®, St. Louis, MO, USA). Chikungunya virus (CHIKV, Strain no. 061573) was used for this study, The virus was propagated in Vero CCL 81 cell line. A 0.01 Multiplicity of Infection (MOI) was used for all experiments.

### Compound stock preparation

The drugs obtained from Sigma Aldrich, St. Louis, MO, USA were included in this investigation. Stock solutions (20 mM) for the drugs were made using dimethyl sulfoxide (DMSO, 100%) as a diluent. These stock solutions were stored at − 20 °C for subsequent analyses.

### Cell viability assay

To evaluate the cytotoxic effects of the compounds, Vero CCL 81 cell lines were used, and the 3-(4,5-dimethythiazol-2-yl)-2,5-diphenyl tetrazolium bromide (MTT) reduction assay was employed based on the method described previously [1]. Briefly, we used the MTT reduction assay to assess the potential toxicity of various compounds. Vero CCL 81 cells were cultured in 96-well plates with an initial density of 3.5 × 10^4^ cells per well. Different combinations of compounds were added to the wells, and their concentrations were determined based on previous experiments to ensure they were not toxic when used individually. The plates were then incubated for 48 h. After incubation, MTT solution (5 mg/mL) was added and allowed to incubate for an additional 3 h. The culture medium was removed, and each well was treated with 100 µL of acidified isopropanol (5% 0.1 N HCl in isopropanol), followed by a 1-hour incubation at 37 °C. Finally, a microplate reader (BioTek Synergy, Santa Clara, CA, USA) was used to measure the absorbance at 570 nm with a reference filter at 690 nm for all the different drug combinations and concentrations tested.

### Antiviral assay

To evaluate the effectiveness of the drug combinations against CHIKV, we selected the concentrations that demonstrated ≥ 90% cell viability, indicating non-toxicity. For the anti-CHIKV activity screening, 2 × 10^5^ cells/well were seeded and allowed to form a complete monolayer for 24 h. The cells were then infected with the virus at a MOI of 0.01 in 100µL (MOI was calculated based on the number of cells used for seeding). After one hour of virus adsorption, the inoculum was removed, and the cells were washed twice with PBS. The drug combinations were added to the wells and incubated for 24 h. Following infection, the culture supernatants were subjected to freeze-thaw cycles and analyzed for virus titre and viral RNA copy number using focus forming unit (FFU) assay and real-time reverse transcription polymerase chain reaction (RT-PCR), respectively. A virus control (VC), consisting of infected cells without any treatment, was maintained in all conditions. Combinations that exhibited enhanced antiviral activity compared to the individual drugs were selected for further dose-dependent studies.

### Quantitative RT-PCR

For the RT-PCR assay, CHIKV-specific primers and probes were used, and viral RNA load was quantified using a standard graph generated from viral RNA standards. The assay was performed in triplicates. For real-time RT-PCR assay, the CHIKV specific primers and probes and the PCR cycle conditions have been provided as earlier [12]. The quantification of viral RNA in the samples was determined by comparing the results to a standard graph. This graph was generated by diluting viral RNA standards and subjecting them, along with the tested samples, to real-time reverse transcription polymerase chain reaction (RT-PCR). All the experiments were performed in triplicates. Virus titers were expressed in log_10_ titers and compared between different treatment groups using one-way ANOVA. The P values were corrected for multiple comparisons.

### Focus forming unit assay

FFU assay was conducted in 96- well plate using Vero CCL-81 cells [12]. Cells were seeded at a density of approximately 3.5 × 10^4^ cells per well and incubated for 24 h. The supernatant from cells treated with different drug combinations was serially diluted and used to infect pre-seeded plates. After one hour of incubation, MEM containing 2% FBS and 1.8% carboxymethyl cellulose was added, and the plates were further incubated for 24 h. The cells were then fixed, blocked and incubated with primary and secondary antibodies. True Blue peroxidase substrate was used to visualize the infected foci, which were counted to determine the virus tire.

### Immunofluorescence assay

To find out the effect of different drug combinations on the infectivity of cells by CHIKV, immunofluorescence assay was performed as described earlier [12].

In this experiment, Vero CCL-81 cells were seeded onto coverslips in a 24-well plate at a density of 2 × 10^5^ cells per well. After 24 h, the cells formed a confluent monolayer and were infected with CHIKV at a MOI of 0.01. Following infection, different concentrations of the drug combinations were added to the wells and incubated for 24 h at 37 °C in a CO2 incubator. To visualize the infected cells, the cells attached to the coverslips were fixed using a mixture of methanol and acetone (1:1 ratio) for 40 min. After fixation, the cells were blocked with 1% bovine serum albumin (BSA) and washed with phosphate-buffered saline containing Tween 20 (PBST). The cells were then incubated with an in-house prepared anti-CHIKV monoclonal antibody (ClVE4/D9 clone) at a dilution of 1:50. This was followed by incubation with a secondary antibody, anti-mouse IgG conjugated with FITC, at a dilution of 1:50 for 40 min. The coverslips were mounted on slides using a mounting solution called mowiol, which contained a nuclear stain called 4’,6-diamidino-2-phenylindole, dihydrochloride (DAPI). The slides were examined using an EVOS Floid cell imaging station at 20x magnification. The fluorescent images were analyzed using ImageJ software. In each coverslip, four to five randomly selected fields were used to count infected and uninfected cells. The average percentage of infected cells was calculated for further analysis.

### Western blot

In the western blotting assay, Vero CCL-81 cells were infected with CHIKV and treated with different concentrations of drugs [1]. Following the 24-hour incubation period, the cells were lysed using RIPA buffer, and the lysates were subjected to electrophoresis on 10% SDS polyacrylamide gels. The proteins were then transferred onto nitrocellulose membranes. To prevent non-specific binding, the membranes were blocked with a solution of phosphate-buffered saline (PBS) containing bovine serum albumin. Subsequently, the membranes were incubated overnight with an anti-CHIKV monoclonal antibody (mAb) specific to the capsid (C) protein. After thorough rinsing to remove any unbound antibody, the membranes were incubated with a secondary antibody conjugated to horseradish peroxidase (HRP). The blots were developed using True Blue peroxidase substrate, and the resulting images were captured using a Gel Documentation system. To quantify the protein signals on the blots, ImageJ software was used. The relative levels of viral protein were normalized to a housekeeping protein, beta-actin, which was detected using a monoclonal antibody (15G5A11/E2, catalog no: MA1-140, Invitrogen, USA). Signal densities were determined, background signals were subtracted, and the data were analyzed and plotted using GraphPad Prism software. This assay enabled the detection and quantification of viral protein expression under different treatment conditions.

### Statistical analysis

The experiments were conducted in triplicate, repeated over two to three independent trials. The percentage viability of the cells at various drug concentrations was calculated by comparing them to the vehicle control-treated cells. The CC50 values (concentration causing 50% cell death) of the drugs were determined using non-linear regression analysis with the aid of GraphPad Prism software version 8.

## Results

### Effect of drug combination on the cell viability

The cytotoxic effects of the drugs combinations with different concentrations of each drug (n = 15) were investigated using 3-(4,5-dimethylthiazol-2-yl)-2,5-diphenyl-2 H-tetrazolium bromide (MTT) assay in Vero CCL-81. The percentage of viable cells at various concentrations of drugs in each combination compared to the cell control was calculated and the drug concentrations that allowed for approximately more than 90% cell viability in each combination were used for studying antiviral activity. For primary screening the maximum non-toxic doses of different drugs were selected in each combination. The effect of different combinations on cell viability is represented as percent of cell viability in Supplementary Figure S1.

### Screening of combinations of the candidate repurposed drugs for improved antiviral activity

**Fig. 1:**
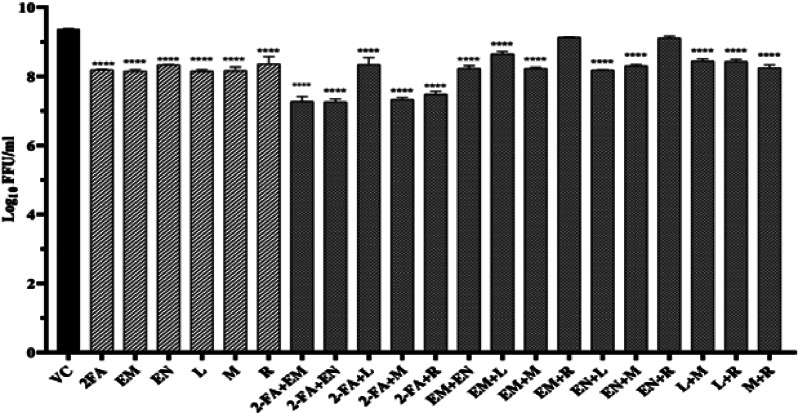
Evaluation of combined repurposed drugs against CHIKV using FFU assay under post-treatment conditions. (**a**) Screening of individual and combination of drugs at their maximum no-toxic concentration for anti-CHIKV activity under post treatment conditions

For evaluating the antiviral activity, monolayer of Vero CCL81 cells were infected with 0.01 multiplicity of infection and different combinations containing maximum non-toxic dose of each drug were added to the cultures and incubated for 24 h post infection. After incubation, the virus titre in the culture was assessed by focus forming unit (FFU) assay as described earlier [1]. Out of the 15 drug combinations tested, three combinations 2FA + EM, 2FA + EN, 2FA + M exerted a reduction of 2 log titre of CHIKV compared to virus control. Ten combinations exerted 1 log reduction in viral titre while the two combinations did not show any significant virus reduction (Table 1 and Fig. 1). Three combinations which showed more than 2 log reduction in virus titre were further selected for dose dependent antiviral studies.

**Table 1:** Log differences in the CHIKV titre compared to virus control on treatment with individual and combination of drugs under post treatment conditions

Sr. no.	Drug name	Conc. (µM)	Log diff.	Combination Drugs	Conc. (µM)	Log diff.
1	2-FA	100	1.184	2-FA + Emetine 2-FA + Enalprilat 2-FA + Lomibuvir 2-FA + Metyrapone 2-FA + Resveratrol	100 + 200 100 + 1.56 100 + 6.25 100 + 200 100 + 12.5	2.10 2.11 1.00 2.04 1.89
2	Emetine	200	1.215	Emetine + Enalprilat Emetine + Lomibuvir Ementine + Metyrapone Emetine + Resveratrol	100 + 1.56 100 + 6.25 100 + 200 100 + 12.5	1.14 0.72 1.14 0.24
3	Enalprilat	100	1.08	Enalprilat + Lomibuvir Enalprilat + Metyrapone Enalprilat + Resveratrol	1.56 + 6.25 1.56 + 200 1.56 + 100	1.19 1.07 0.26
4	Lomibuvir	6.25	1.215	Lomibuvir + Metyrapone Lomibuvir + Resveratrol	6.25 + 200 6.25 + 12.5	0.93 0.94
5	Metyrapone	100	1.203	Metyrapone + Resveratrol	200 + 12.5	1.1204
6	Resveratrol	12.5	1.01			

### Dose dependent antiviral activity of combination of drugs against CHIKV

**Fig. 2:**
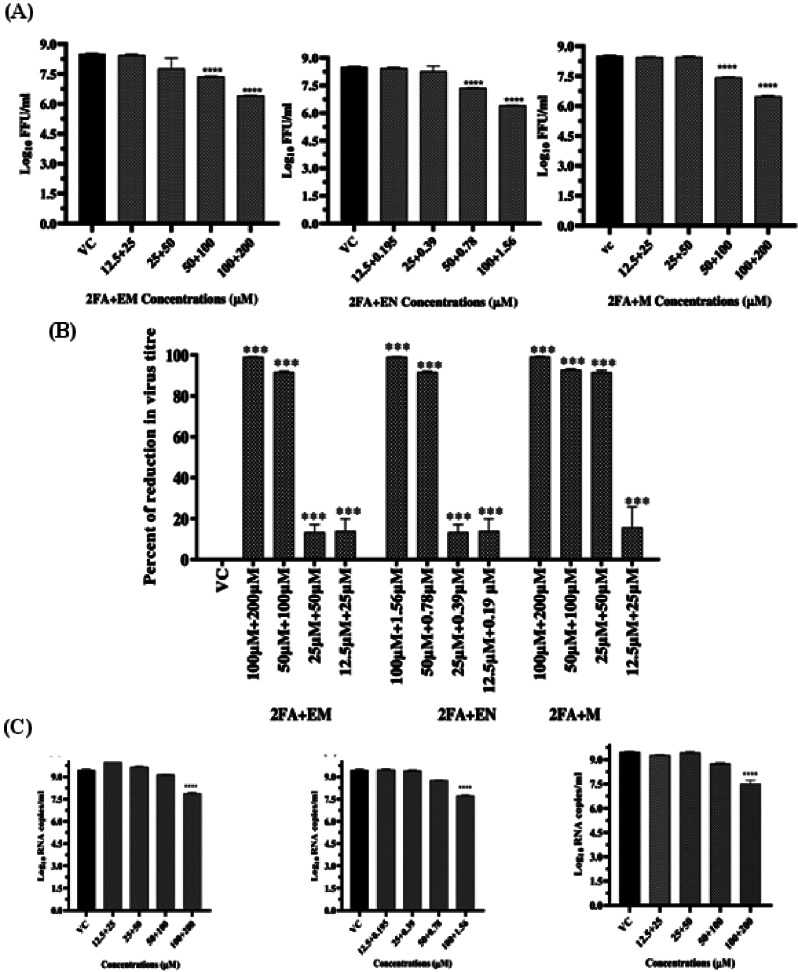
(**A**) Dose dependent antiviral effect of all effective drug combinations on CHIKV titre. Highest non-toxic concentrations of 2FA + EM, 2FA + EN, 2FA + M combinations showed a 2 log virus reduction and at half the highest non-toxic concentration, 1 log virus reduction was observed compared to VC. (**B**). Percent virus reduction exerted by effective drug combinations. The percent reduction was calculated using virus control as reference. In all cases, comparisons were made between drug treatments and VC. All data was presented as mean ± SE of three individual experiments. one-way ANOVA with multiple comparisons was used to find out the statistical significance****p < 0.0001, *** p < 0.0004. (**C**) Evaluation of combined repurposed drugs against CHIKV using qRT-PCR assay under post-treatment conditions. The graph represents the viral copy number. The viral copy number in the treated group was significantly reduced when compared with the control group

To investigate the dose dependent antiviral effect of the three combinations that showed a significant reduction in virus titre under post-infection treatment conditions, the concentrations of the drugs in each combination were diluted with medium in the ratio of 1:1 up to three more concentrations and the antiviral activity was evaluated. The virus output after the antiviral assay was evaluated by measuring infectious virus titre by FFU assay, viral RNA copy number by real-time RT-PCR, percent infected cells by immunofluorescence assay and viral protein expression by western blotting as described earlier [1]. FFU assay results indicate a dose dependent reduction in virus titre. The reduction in FFU/ml in infected cultures treated with each combination of drugs compared to virus control was significant in cultures treated with maximum and half the highest non-toxic concentration of different drug in each combination (Fig. 2A). The percent reduction was ~ 99% in infected cultures treated with maximum non-toxic dose of different drugs in each combination while it was ~ 90% in infected cultures treated with half the highest non-toxic concentration of different drug in each combination (Fig. 2B). FFU results were validated using RT-PCR analysis. The maximum non-toxic dose of each drug in each combination significantly reduced viral RNA copy number in infected cultures (Fig. 2C).

### Reduction in the infected cells

**Fig. 3:**
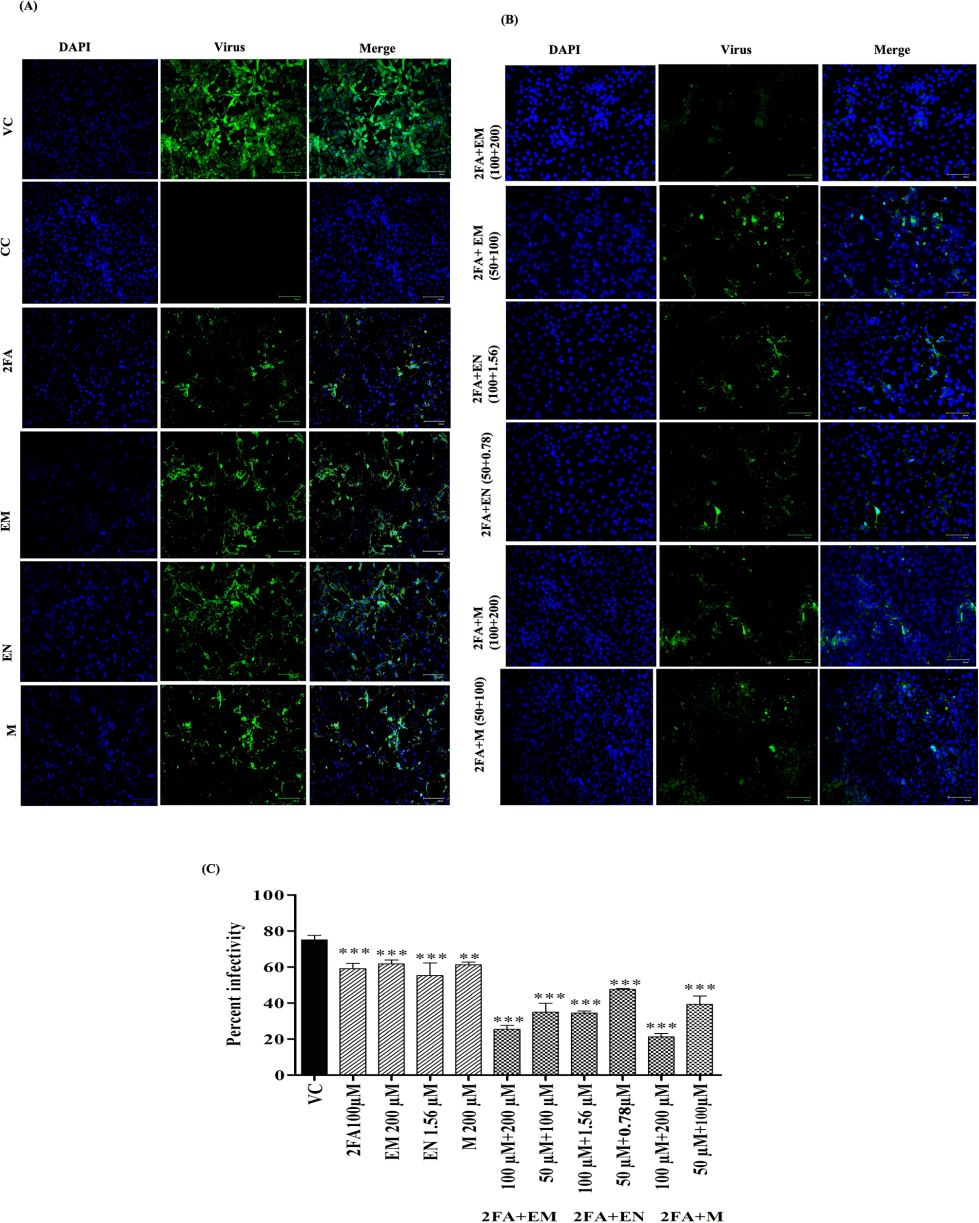
Effect of combined repurposed drugs on CHIKV infection under posttreatment condition using IFA (A) Immunofluorescence assay images depict CHIKV-infected Vero CCL-81 treated with different concentrations of individual compounds under posttreatment conditions are presented where virus-infected cells are stained with FITC depicted in green, while cell nuclei, stained with DAPI, are depicted in blue. The merged image is also included for comprehensive visualization. 2FA 100µM, EM 200µM, EN 1.56µM, M 200µM (B) Immunofluorescence assay images of CHIKV infected Vero CCL-81 cells treated with different concentrations of combined compounds under post treatment conditions are presented. 2FA + EM 100µM + 200µM, 2FA + EM 50µM + 100µM, 2FA + EN 100µM + 1.56µM, 2FA + EN 50µM + 0.78µM, 2FA + M 100µM + 200µM, 2FA + M 50µM + 100µM. (C) The mean percentage of infected cells was calculated relative to the total number of cells. The values were expressed as mean ± standard error (SE) of triplicates. The virus-infected cells are shown in green and percent of infected cells is plotted on the graph

The results of the immunofluorescence assay (IFA) indicated a significant reduction in the percentage of infected cells when treated with the combination of repurposed drugs compared to both individual drugs and the vehicle control (VC) group. In Fig. 3A and 3B, the data obtained from the IFA results are presented. These figures demonstrate the effects of the drug combinations on the infection rate of CHIKV in cells. The percent of infected cells was shown to be markedly decreased when treated with the combination therapies, indicating their anti-CHIKV activity. These findings suggest that combining the repurposed drugs (2FA + EM, 2FA + EN, and 2FA + M) leads to an additive effect in inhibiting CHIKV infection compared to the use of each drug individually or the control group. The immunofluorescence assay results provide visual evidence of the reduced infection rate, reinforcing the potential therapeutic value of these drug combinations in the context of CHIKV infection.

### Reduction in envelope/capsid protein expression

**Fig. 4:**
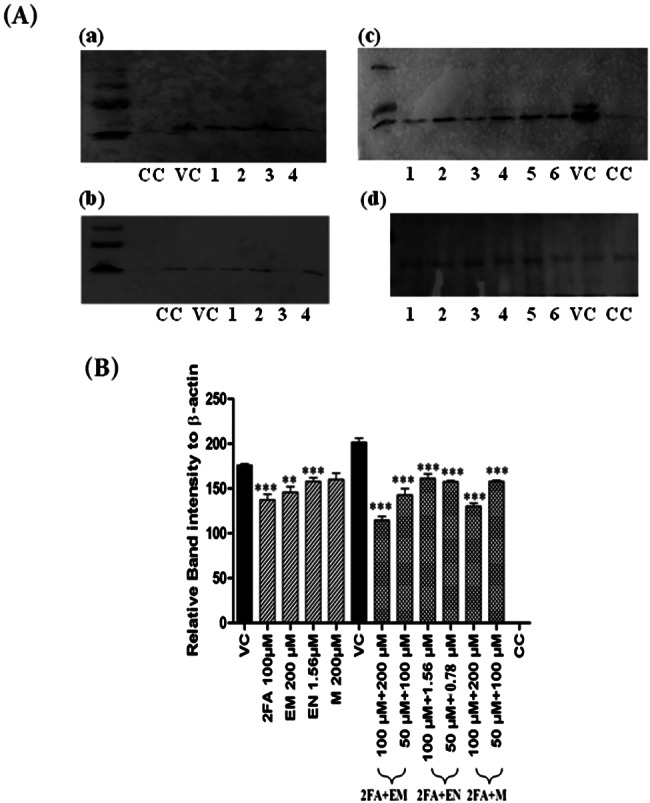
(A) Western blot images were shown, demonstrating variations in viral protein expression in cultures treated with individual drugs or their combinations. The blots for individual drugs included (a) envelope protein expression and: Lane-1: 2FA 100µM, Lane-2: EM 200µM, Lane-3: EN 1.56µM, and Lane-4: M 200µM. The blots for combined drugs (c) Lane-1: 2FA + EM 100µM + 200µM; Lane-2: 2FA + EM 50µM + 100µM; Lane-3: 2FA + EN 100µM + 1.56µM; Lane-4: 2FA + EN 50µM + 0.78µM; Lane-5: 2FA + M 100µM + 200µM; Lane-6: 2FA + M 50µM + 100µM. (b &d) β-actin expression. VC: Virus control CC: Cell control PM: Protein marker. (B) ImageJ software was used to analyze the band density intensities of the envelope antigens (mean ± SE, n = 3 replicates). Lane-1, 3, and 5 exhibited a significant reduction in envelope antigens compared to VC. Statistical analysis was performed using one-way ANOVA with multiple comparisons against the viral control, indicated by *** p < 0.0004. The combination drugs (d) Shows RT-PCR results, a higher percentage of viral reduction compared to individual drugs. The graph compares the different concentrations combination drugs with the viral control. All values are expressed as mean ± SE of three individual experiments. Statistical analysis was performed using one-way ANOVA multiple comparisons with viral control and indicated by *** p < 0.0004

The western blot analysis was conducted to study the effect of the drug combinations (2FA + EM, 2FA + EN, and 2FA + M) on CHIKV protein expression. A cell lysate obtained from CHIKV-infected Vero cells and CHIKV-infected treated cells underwent electrophoresis in a 10% SDS-PAGE gel. CHIKV antigen probed with anti-C mAb revealed a band at 31 kDa, corresponding to the C protein of CHIKV. CHIKV antigen probed with a viral control (VC) for CHIKV displayed bands at 31–52 kDa, corresponding to the C protein of CHIKV. CHIKV antigen probed with a negative control (CC) showed no reaction. Specifically, the analysis focused on the expression of the capsid protein of the virus in cell cultures treated with the drug combinations, individual drugs, and a viral control (VC) group. The data in these figures demonstrate a significant reduction in the expression levels of the capsid protein in the cultures treated with the combination of repurposed drugs compared to both the individual drugs and the VC group. Western blot analysis revealed a significant reduction in the expression of capsid protein of virus in cultures treated with combination of repurposed drugs compared to individual drugs and VC group (Fig. 4 iii and iv). Full images of the blots were shown in the Supplementary Figure S2.

## Discussion

Given the severity and widespread nature of Chikungunya fever, the development of effective inhibitors against CHIKV is of utmost importance. While several antiviral compounds have been explored, most are still in the early stages of drug development and have only been tested in vitro [20]. Repurposing approved drugs for anti-CHIKV treatment could provide a fast and safe option, as their safety profiles in humans are well-established [21]. In our previous studies, we identified FDA-approved drugs with demonstrated anti-DENV and anti-CHIKV activity using a systems biology approach and in vitro studies [1, 12]. Utilizing combinations of effective drugs might lead to synergistic or additive antiviral effects and might also help to reduce the drug dosage and emergence of drug resistance [2]. Hence, in the present study, antiviral effects of 15 combinations of six drugs, 2-fluoroadenine (2-FA), lomibuvir (L), emetine (EM), enalaprilat (EN), metyrapone (M), and resveratrol (R) were explored against CHIKV. The study revealed that 2FA + EM, 2FA + EN, 2FA + M combinations exerted two log reduction in CHIKV titre in a dose dependent manner which was confirmed by measuring the titre in terms of infectious virus particles, viral RNA copy number, percent cells infected and viral protein expression using different assays. The antiviral effect was additive in nature while combination of drugs was used compared to single drugs. The additive effects observed could be due to the different viral targets affected by the drugs. Our earlier in-silico studies have revealed that 2-FA showed highest binding affinity with the CHIKV NSP3 protein while EM and EN showed the highest binding affinity with the CHIKV NSP2 helicase and protease respectively. Metyrapone interacted effectively with the envelope glycoprotein [1]. These different targets indicated for each drug may explain the additive effects of these combinations against CHIKV [1]. Moreover, it also has been shown that these drugs when tested individually, affected the CHIKV at different time points of treatment with respect to infection. 2-FA exerted greatest inhibitory activity against CHIKV under both pre- and post-treatment conditions, metyrapone and enalaprilat showed best antiviral activity under pretreatment and cotreatment conditions respectively, while emetine under post treatment conditions [1]. Thus, by combining different drugs acting at different time points with respect to infection, it was possible to achieve greater reduction under post infection treatment conditions. This approach is advantageous, however only a limited number of compounds have been tested against CHIKV in animal models, with few specifically designed to target the alphavirus. Several drugs, including suramin, favipiravir, sofosbuvir, pimozide, auranofin and ribavirin (used alone or combined) have shown effectiveness in reducing CHIKV pathological signs in vivo [21–23]. Additionally, compounds such as berberine, and DFMO have demonstrated potential by reducing viral titers by targeting host factors like TOFA, HS-10, SNX-2112, PACMA31,confirming their potential as host targets for anti-CHIKV drug development [20, 24]. Meanwhile, compounds specifically designed to inhibit the alphavirus, such as bis(benzofuran-thiazolidone) (3 g), MADTP (9b), CHVB-032, compound 25, compound 8, and compound-A, have exhibited promising antiviral activity in vitro [25]. Emerging and re-emerging viruses can be treated more effectively by combination of drugs that exert additive or synergistic antiviral effects [26]. Trials combining ribavirin and interferon in an in vitro model and on animals have shown promising results in reducing CHIKV titre as compared to monotherapy with ribavirin alone [27]. Another study demonstrated a 91% reduction in viral replication when treating CHIKV with the combination of Doxycycline and Ribavirin compared to individual treatments [28]. The combination treatment using epigallocatechin-3-gallate and suramin inhibited CHIKV replication and reduced viral load in infected cells, showing a synergistic effect. The treatment was effective against multiple strains of CHIKV [29]. The combination treatment of mefenamic acid and ribavirin showed significant antiviral activity against CHIKV in vitro and in vivo. The treatment reduced viral load, inflammation and joint swelling in infected mice [30]. While our study provides promising insights and might serve as a basis for an innovative therapeutic approach to treat patients infected with CHIKV, we acknowledge certain limitations. Further validation through animal models and clinical trials is warranted to confirm the efficacy and safety of these drug combinations, paving the way for the development of efficient treatment regimens.

## Conclusion

In conclusion, the study demonstrated that the combination of 2-fluoroadenine, emetine, enalaprilat and metyrapone exhibited superior efficacy compared to individual drug treatments. The findings suggest that combination therapies have the potential to be more effective in treating CHIKV and other emerging and re-emerging viruses. Overall, this research paves the way for potential advancements in the treatment of CHIKV-infected patients.

### Electronic supplementary material

Below is the link to the electronic supplementary material.

Supplementary Material 1: Effect of repurposed drugs on Vero CCL-81 cells and represents the mean percentage of viable cells relative to untreated control cells. The average of three replicates with SE is represented by each bar The x-axis represents the individual’s single highest non-toxic concentration and four highest non-toxic concentrations of combination drugs

Supplementary Material 2: (a) Western blot images were presented, illustrating changes in viral protein expression in cultures treated with individual drugs or combinations. For the individual drug blots, they included the following conditions: Lane-1: 100µM of drug 2FA, Lane-2: 200µM of drug EM, Lane-3: 1.56µM of drug EN, and Lane-4: 200µM of drug M. (Lane 5,6 were duplicates in the uncropped blot) (b). As for the combined drug blots, they were as follows: Lane-1: 100µM of 2FA + 200µM of EM, Lane-2: 50µM of 2FA + 100µM of EM, Lane-3: 100µM of 2FA + 1.56µM of EN, Lane-4: 50µM of 2FA + 0.78µM of EN, Lane-5: 100µM of 2FA + 200µM of M, and Lane-6: 50µM of 2FA + 100µM of M. (b, d) Additionally, the blots included β-actin expression as a control, VC: Virus control CC: Cell control PM: Protein marker

## Data Availability

Data will be made available on request if necessary.

## Abbreviations

MTT: 3-(4,5-dimethythiazol-2-yl)-2,5-diphenyl tetrazolium bromide
CHIKV: Chikungunya
NSP: Non structural protein
