# Supplementary figures and images for: Repurposed drugs in combinations exert additive anti-chikungunya virus activity: an in-vitro study
Source: Virol J. 2024 Jan 4;21:5. doi: 10.1186/s12985-023-02271-0 (PMC10768230; doi:10.1186/s12985-023-02271-0)

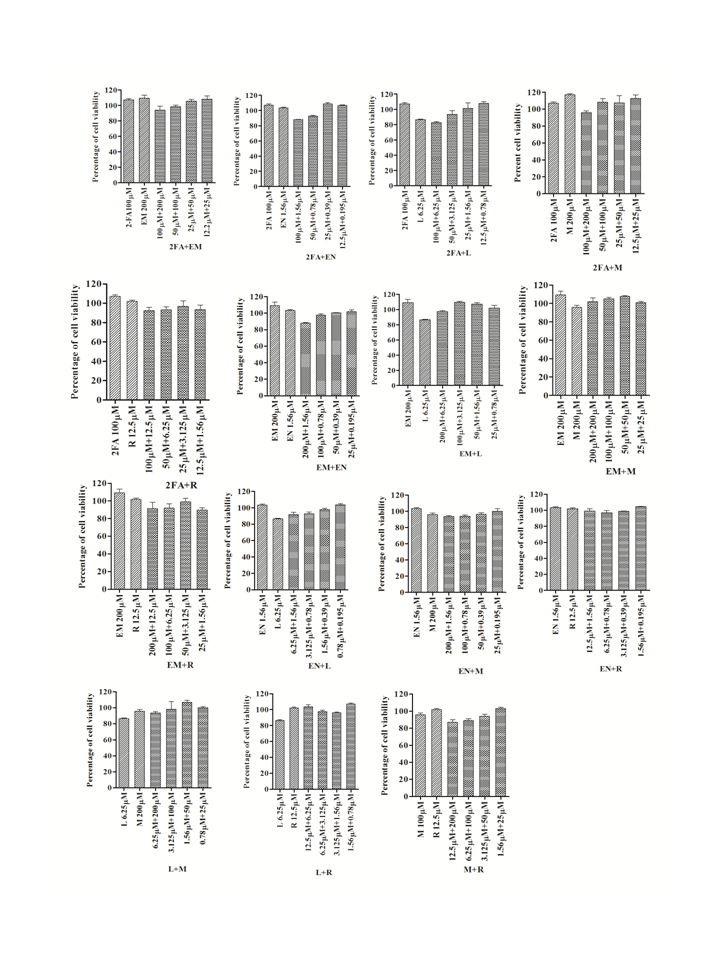

Supplement: Supplementary file 1 — Supplementary Material 1: Effect of repurposed drugs on Vero CCL-81 cells and represents the mean percentage of viable cells relative to untreated control cells. The average of three replicates with SE is represented by each bar The x-axis represents the individual’s single highest non-toxic concentration and four highest non-toxic concentrations of combination drugs [file 12985_2023_2271_MOESM1_ESM.tiff]

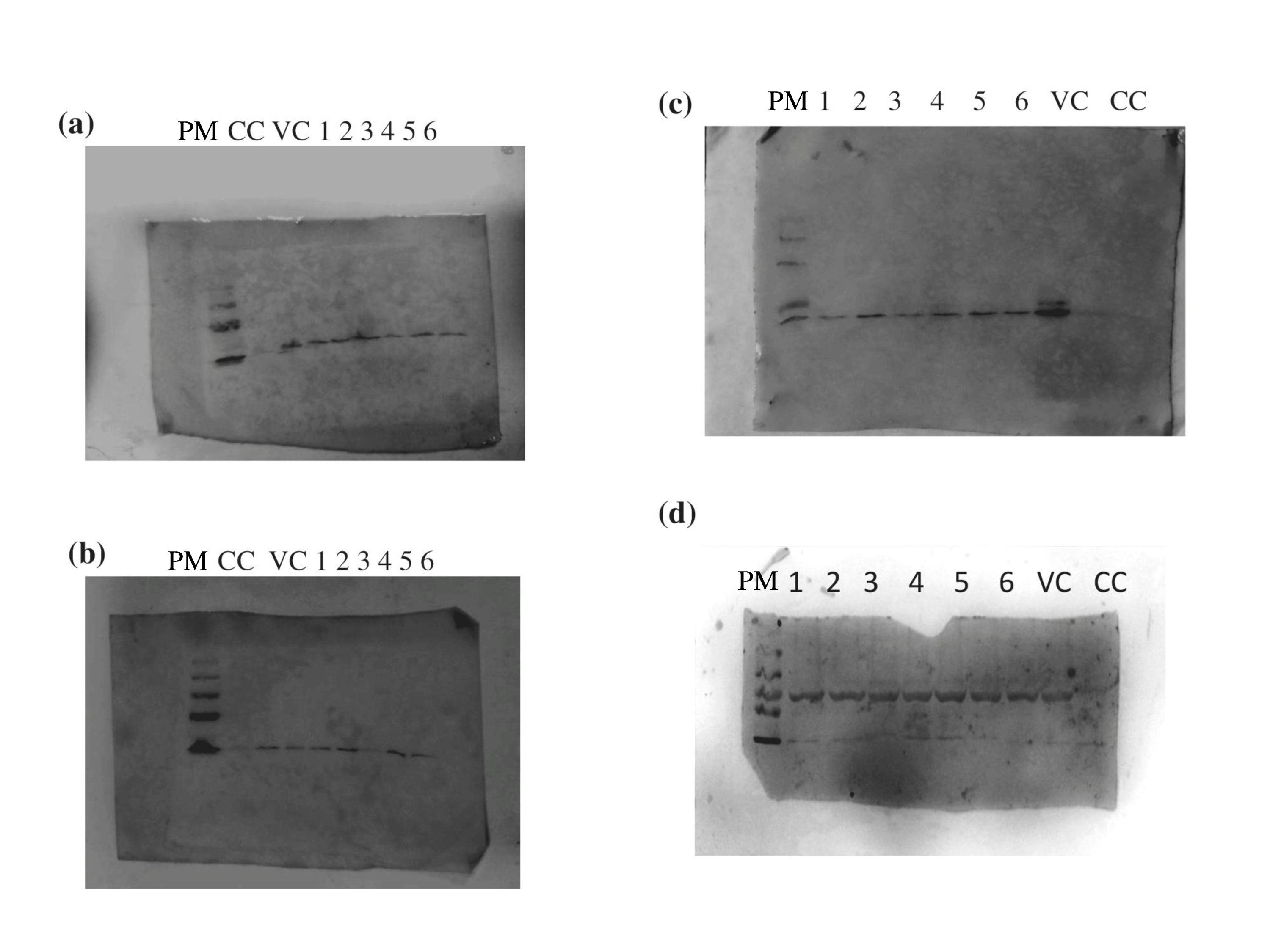

Supplement: Supplementary file 2 — Supplementary Material 2: (a) Western blot images were presented, illustrating changes in viral protein expression in cultures treated with individual drugs or combinations. For the individual drug blots, they included the following conditions: Lane-1: 100µM of drug 2FA, Lane-2: 200µM of drug EM, Lane-3: 1.56µM of drug EN, and Lane-4: 200µM of drug M. (Lane 5,6 were duplicates in the uncropped blot) (b). As for the combined drug blots, they were as follows: Lane-1: 100µM of 2FA + 200µM of EM, Lane-2: 50µM of 2FA + 100µM of EM, Lane-3: 100µM of 2FA + 1.56µM of EN, Lane-4: 50µM of 2FA + 0.78µM of EN, Lane-5: 100µM of 2FA + 200µM of M, and Lane-6: 50µM of 2FA + 100µM of M. (b, d) Additionally, the blots included β-actin expression as a control, VC: Virus control CC: Cell control PM: Protein marker [file 12985_2023_2271_MOESM2_ESM.tiff]
